# Congenital Cytomegalovirus and Human Immunodeficiency Virus: Effects on Hearing, Speech and Language Development, and Clinical Outcomes in Children

**DOI:** 10.3389/fped.2021.771192

**Published:** 2021-12-16

**Authors:** Hannah Walsh, Jillian Zuwala, Jessica Hunter, Yonghee Oh

**Affiliations:** Department of Speech, Language, and Hearing Sciences, University of Florida, Gainesville, FL, United States

**Keywords:** congenital cytomegalovirus, human immunodeficiency virus, hearing, speech and language development, clinical outcomes in children

## Abstract

Prenatal infections can have adverse effects on an infant's hearing, speech, and language development. Congenital cytomegalovirus (CMV) and human immunodeficiency virus (HIV) are two such infections that may lead to these complications, especially when left untreated. CMV is commonly associated with sensorineural hearing loss in children, and it can also be associated with anatomical abnormalities in the central nervous system responsible for speech, language, and intellectual acquisition. In terms of speech, language, and hearing, HIV is most associated with conductive and/or sensorineural hearing loss and expressive language deficits. Children born with these infections may benefit from cochlear implantation for severe to profound sensorineural hearing losses and/or speech therapy for speech/language deficits. CMV and HIV simultaneously present in infants has not been thoroughly studied, but one may hypothesize these speech, language, and hearing deficits to be present with potentially higher severity. Early identification of the infection in combination with early intervention strategies yields better results for these children than no identification or intervention. The purpose of this review was to investigate how congenital CMV and/or HIV may affect hearing, speech, and language development in children, and the importance of early identification for these populations.

## Introduction

There are many infectious diseases that can adversely affect auditory system development if a fetus is exposed to the infection *in-utero*, during delivery, or shortly after birth. These include rubella, Zika virus, lymphocytic choriomeningitis virus (LCMV), herpes simplex virus (HSV) types 1 and 2, measles, varicella zoster virus, mumps, and West Nile virus (1). Additionally, cytomegalovirus (CMV) and human immunodeficiency virus (HIV) are viral infections that may cause hearing loss and neurodevelopmental deficits in a developing fetus, leading to hearing and speech complications in childhood and early adolescence. CMV is the “leading cause of (congenital) non-hereditary sensorineural hearing loss (SNHL) in the developed world” (2), affecting approximately 1 in every 100–200 births (3). Ten percent of these infants are symptomatic at birth (2, 3). HIV has infected 36.7 million people in the world, with 17.8 million cases being women of childbearing age, and approximately 2.2 million cases being children (4). Previous research suggests children with congenital hearing loss due to environmental and/or genetic factors may have delayed speech and language development if not adequately diagnosed by 6 months of age (3). Since CMV is the number one cause of virus-induced congenital hearing loss and HIV is well-known to the general public, the current research review investigates how congenital CMV and/or HIV may affect hearing, speech, and language development in children, and the importance of early identification for these populations.

## Methods

### Search Strategy

The search was conducted by the first author from April 2021 to July 2021. Each source was located through PubMed and was refined to a publication date from 1990 to 2021 to find the most up-to-date literature. The topics relate to two common prenatal infections (CMV and HIV) and their relationship to hearing loss and speech/language delays in infants. The following four search strategies were utilized to obtain all of the sources for this paper, as listed below:

(cytomegalovirus OR CMV) AND (hearing loss OR speech OR language) AND (child^*^ OR pediatric^*^)
(congenital cytomegalovirus OR human cytomegalovirus) AND (magnetic resonance imaging OR MRI)
(HIV) AND (hearing loss OR speech OR language) AND (child^*^ OR pediatric^*^)
(HIV OR human immunodeficiency virus OR herpes simplex OR AIDS OR congenital HIV) AND (sensorineural hearing loss OR maternal transmission OR cochlea OR neurocognit^*^ OR neuropath^*^ OR otolog^*^ OR cerebrum)

### Selection of Studies

**Figure 1:**
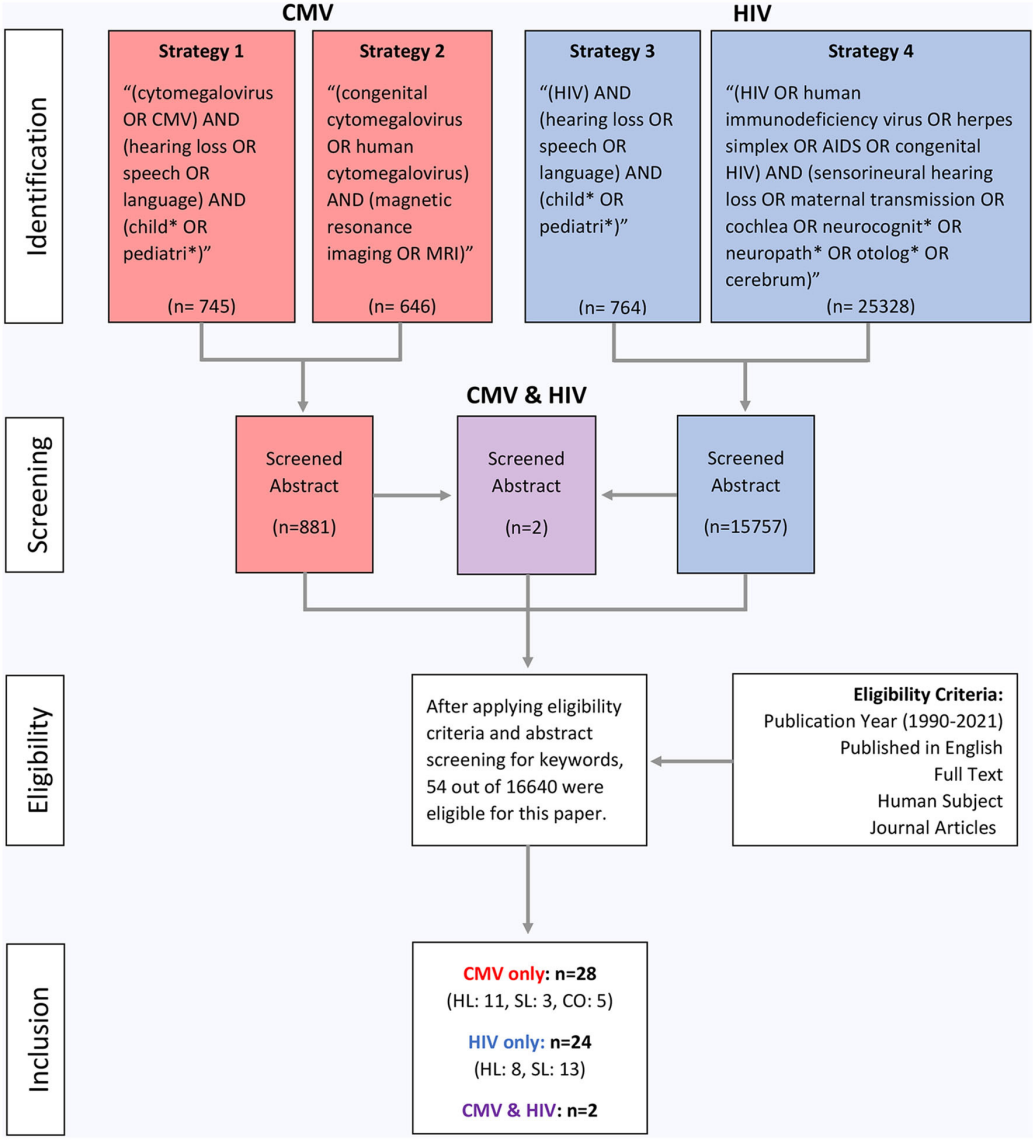
Flow diagram of study inclusion. CMV denotes cytomegalovirus, HIV denotes human immunodeficiency virus, MRI denotes magnetic resonance imaging, HL denotes hearing loss, SL denotes speech/language, and CO denotes clinical outcomes.

A number of filters were added to narrow the search results for each of the search strategies, including publication year (1990–2021), published in English, full text, human subject, and journal articles. The following age filters were also applied: child (birth-18 years), infant (birth-23 months), infant (1–23 months), newborn (birth-1 month), and preschool children (2–5 years). Search strategy 4 did not include these age filters in order to find detailed information pertaining to general HIV infection on anatomical structures. Specific search strategy information is described in Figure 1.

## Discussion

### Cytomegalovirus (CMV)

Researchers have investigated how congenital CMV (cCMV) (Table 1) is passed from mother to child during pregnancy, as well as the implications of the virus on the developing fetus. Foulon et al. (2) studied two modes of cCMV transmission (i.e., primary and non-primary) and the risk factors for SNHL in children with cCMV. A mother with primary CMV is one who tests positive for CMV during pregnancy, whereas a mother with non-primary CMV tests positive for CMV antibodies during pregnancy, indicating prior infection and current immunity (2). Whether there is primary or non-primary CMV infection, there can be adverse effects on an infant's hearing and neurodevelopment. Giannattasio et al. (8) compared the infants of 93 mothers with primary CMV infection to the infants of 65 mothers with non-primary CMV infection, finding no statistically significant difference in the prevalence of hearing and neurodevelopmental concerns between the two groups. Similarly, Foulon et al. (2) studied 157 children with cCMV and found 76 infants were born to mothers with primary CMV and 42 infants were born to mothers with non-primary CMV. These newborns were found to have cCMV based on virological screening results from saliva or urine samples collected within 5 days after birth (2).

**Table 1:** Background of CMV and effects of CMV on hearing, speech and language development, and clinical outcomes in children.

	**References**	**Study design**	**Population**	**Major findings**	**Search strategy**
Background (*n* = 9)	Foulon et al. (5)	Observational Study	28 children born with cCMV whose mothers had a primary CMV diagnosis	Trimester in which the mother's primary CMV infection occurred was estimated and all children were screened for SNHL. Results showed that SNHL is more common in cCMV-positive infants born to mothers who became infected with CMV in their first trimester than mothers infected later in pregnancy.	1
	Foulon et al. (2)	Observational Study (Prospective Longitudinal Study)	157 children with cCMV, identified within 5 days of birth	Children with cCMV may have certain risk factors (infection before the 14th week of pregnancy, infection at birth, and imaging abnormalities) for SNHL.	1
	Fowler and Boppana (6)	Systematic Review	Mothers with CMV, babies with cCMV	There is little awareness among pregnant women and healthcare providers about cCMV and associated risks. Prenatal testing for CMV generally only occurs as part of the diagnostic evaluation for mononucleosis and similar illnesses. CMV screening technologies for newborns have improved and CMV-related SNHL is more likely to be detected thanks to routine newborn hearing screenings. Newborn CMV screenings themselves are not routine, however. Longer-term antiviral treatments for infants with symptomatic cCMV are beneficial.	1
	Fowler et al. (7)	Proposal	99,945 newborns screened for cCMV at 7 different medical centers between 2007 and 2012	Infants were screened for cCMV, along with their routine newborn hearing screening, while in the nursery. 7% of CMV-positive newborns failed their newborn hearing screenings, compared to only 0.9% of newborns who were not cCMV positive. Most cCMV-positive newborns who failed their hearing screening had SNHL. A targeted approach to test all newborns who fail their hearing screenings for cCMV is proposed. It is important to note that 43% of infants with CMV-related SNHL were not identified by the newborn hearing screening.	1
	Giannattasio et al. (8)	Cohort Study	158 congenitally infected children; 93 born to mothers with primary CMV infection and 65 with non-primary infected mothers	The frequency of hearing loss and neurodevelopmental deficits in newborns does not vary with type of maternal CMV infection.	1
	Kimberlin et al. (9)	Randomized Control Trial	2 randomly assigned groups of neonates with CMV disease involving the central nervous system	One group received 6 weeks of ganciclovir while the other group received no treatment. 21/25 in the ganciclovir group had improved or maintained hearing between baseline and 6 months old. 7/17 control patients had worsening of hearing between baseline and 6 months. Ganciclovir administered in the neonatal period may prevent hearing deterioration.	1
	Nicloux et al. (10)	Review	Newborns with cCMV	cCMV is the most common non-hereditary cause of hearing loss in children. 90% are asymptomatic at birth, and of those 90%, 5–15% are considered at-risk of experiencing neurosensory sequelae, including hearing loss. The 10% of infants who exhibit cCMV symptoms have a much higher risk of neurosensory impairment (17–60%). Even if neurosensory sequelae are not immediately present upon a cCMV diagnosis, they can be delayed, so follow-ups well beyond the neonatal period are recommended.	1
	Shearer et al. (11)	Proposal	Newborns with cCMV	cCMV testing is currently not part of the routine hearing screening a newborn undergoes, despite being one of the major causes for hearing loss in children. This proposal suggests that (limited) genetic testing and cCMV testing are implemented into the universal newborn hearing screening procedures to better identify newborns with hearing loss who may be missed by current testing protocols.	1
	Yamamoto et al. (12)	Secondary Infection Study	11,900 newborns born to mothers in a very seropositive population, of which 68 were cCMV-positive and 91 failed their newborn hearing screening	7/24 (29.2%) of the newborns who failed their hearing screening had cCMV. Integrating targeted cCMV screening among newborns who fail their hearing screening could be a cost-effective strategy to identifying newborns with early-onset, cCMV-related HL.	1
Hearing loss (*n* = 11)	Dar et al. (13)	Cohort Study	1,720 newborns in rural northern India who are CMV-positive and/or failed their newborn hearing screening	cCMV is the leading factor that causes permanent congenital/early-onset hearing loss. Even with nearly universal seroimmunity, there is still a strong correlation between cCMV and hearing loss. This holds true in both the developing world and more rural places, like regions of northern India, and simultaneous cCMV and hearing screenings are both possible and beneficial in a resource-limited setting.	1
	Demmler-Harrison et al. (14)	Longitudinal Study	237 infant-mother pairs with maternal CMV, followed child from birth-18 years of age	Primary and non-primary CMV during pregnancy may result in symptomatic or asymptomatic cCMV. SNHL occurred most commonly after maternal primary infection with CMV. This HL was detected within the first year of life in most cases.	1
	Fletcher et al. (15)	Systematic Review	Children with cCMV-induced SNHL, from 36 articles regarding the history and rehabilitative outcome of cCMV-induced SNHL	9–68% of cCMV-induced SNHL is delayed, meaning it is not uncommon for newborns with cCMV to pass their newborn hearing screenings undetected. In 7–71% of cases, SNHL was progressive. Frequent audiologic evaluation of children who are cCMV-positive is necessary considering the natural history of cCMV-related hearing loss. Because so many cCMV-positive newborns are asymptomatic, universal neonatal screening should be considered.	1
	Kim et al. (16)	Retrospective Case Study	58 children born with cCMV	11/58 (19%) of children with confirmed cCMV infections at birth also had SNHL. Most confirmed HL cases passed their newborn hearing screening and were diagnosed only after re-evaluation following a CMV test. It is important to still perform timely audiologic evaluations of children who are identified to have cCMV, even if they initially passed their newborn hearing screening.	1
	Korver et al. (3)	Systematic Review	children with congenital hearing loss identified at birth due to genetics or infection	Several genetic disorders and infections may result in hearing loss in children. It is important for professionals to focus on early diagnosis and treatment of hearing loss for optimal results.	1
	Lanzieri et al. (17)	Longitudinal Study	92 case patients and 51 controls; assessment was completed with auditory brainstem response and behavioral audiometry	Delayed-onset and progression of SNHL continued through adolescence for children with asymptomatic congenital cytomegalovirus. The risk of developing SNHL after 5 was not different than in uninfected children.	1
	Lanzieri et al. (18)	Longitudinal Prospective Cohort Study	96 case patients of which 4 were symptomatic and 92 asymptomatic.	Congenital/early-onset SNHL frequently resulted in severe to profound HL in both asymptomatic and symptomatic cases.	1
	Riga et al. (19)	Systematic Review	181 children with cCMV-induced hearing loss	The prevalence of cCMV-induced hearing loss was significantly higher among children who were symptomatic and were also much more likely to develop bilateral hearing loss. Infants with cCMV should be closely monitored during their preschool years, regardless of hearing status, since hearing thresholds can change substantially long after the neonatal period.	1
	Rosenthal et al. (20)	Longitudinal Follow-up Study	580 children with cCMV	Urine/saliva samples from 580 children were collected and analyzed for CMV “shedding.” Prevalence of culture positivity decreased significantly after 3 years of age. Delayed HL is strongly associated with symptomatic infection at birth, but many asymptomatic children also developed HL. Longer duration of CMV shedding could predict delayed HL.	1
	Salomè et al. (21)	Prospective Study	102 children with asymptomatic CMV	The long-term audiologic outcomes of 102 children with asymptomatic CMV were measured from 2002 to 2018. Following a mean follow-up period of 3.3 years, none of these 102 children developed SNHL. Only 14 presented with a a variable hearing impairment. Overall, these data suggest that there is a relatively low risk of delayed hearing loss if a cCMV-infected child is asymptomatic during the first month of life.	1
	Verbeeck et al. (22)	Comparative Study	194 infants with indicative hearing impairment, 332 matched controls in Flanders, Belgium	Significantly more infants with hearing impairments were cCMV positive. The presence of CMV before or shortly after birth influences the outcome of hearing impairment. Follow-up study suggests that the hearing impairment of children infected with CMV after birth are less likely to improve than children who are CMV-negative.	1
Speech/ Language (*n* = 3)	De Kegel et al. (23)	Longitudinal Study	64 children with cCMV assessed at 6, 12, and 24 months old	cCMV is a risk factor for early motor development delays.	1
	Lopez et al. (24)	Comparative Study	Children born with cCMV (some of whom have SNHL), and matched controls	cCMV-positive children with SNHL scored significantly lower on full-scale intelligence and receptive vocabulary tests than their normal hearing peers. There were no significant differences among the groups for verbal-nonverbal intelligence, expressive vocabulary, and reading/math achievements.	1
	Zhang et al. (25)	Cohort Study	49 children age birth-6 years in Qinba, China	Asymptomatic cCMV did not relate to physical or intellectual disability, but it may be a predictor of poorer long-term development of language.	1
Clinical outcomes (*n* = 5)	Cannie et al. (26)	Cohort Study	51 fetuses with cCMV; 121 total confirmed patients with cCMV	cCMV can be identified with MRI as early as 27 to 33 weeks gestation. 18 infants showed post-natal SNHL, 10 infants showed post-natal neurological impairment.	2
	Corazzi et al. (27)	Retrospective Case Control Study	Children with both symptomatic and asymptomatic cCMV who received CIs, children the Connexin 26 mutation who received CIs	Connexin 26 mutations are known to cause hereditary bilateral SNHL, and cCMV is known to be the leading cause of non-hereditary SNHL. Children with symptomatic cCMV who received CIs and speech therapy struggled to obtain language more than their asymptomatic cCMV peers and those with Connexin 26 mutations. Regardless, CI usage supported by speech therapy is an excellent intervention plan for cCMV-positive children who suffer from SNHL.	1
	Diogo et al. (28)	Systematic Review	fetuses with cCMV; MRI findings at 29, 31, 34, and 36 gestational weeks	Imaging reveals abnormal brain structures in developing fetuses exposed to CMV, one of the more common abnormalities being lesions in the temporal lobe. Parental and healthcare professional education is recommended, in addition to future research and a solidified screening protocol for prenatal identification of cCMV.	2
	Laccourreye et al. (29)	Cohort Study	15 children with profound HL from cCMV; assessed 3 months pre- and post- cochlear implantation	Children with typical anatomy (as shown on MRI) demonstrated improved hearing and speech production following cochlear implantation. For those with abnormal MRI results, speech therapy and balance rehabilitation were recommended for best outcomes.	1
	Natale et al. (30)	Retrospective Case Study	60 infants born with asymptomatic cCMV, none of whom underwent antiviral treatment	16/60 (26.67%) of infants had some form of auditory neuropathy, mainly moderate. However, all 16 of these infants spontaneously recovered a normal auditory threshold over time. A delayed maturation of the auditory system should be considered when an isolated mild-moderate SNHL is found at birth as a result of cCMV.	1

*cCMV, congenital cytomegalovirus; CMV, cytomegalovirus; HL, hearing loss; MRI, magnetic resonance imaging; SNHL, sensorineural hearing loss*.

However, universal CMV testing is not commonplace in the American healthcare system, usually being implemented only when there is speculation of other viruses, such as mononucleosis (6). One such method of testing for CMV is polymerase chain reaction (PCR)-based sequencing testing, which involves analyzing the genetic components of biological samples. Shearer et al. (11) recommend implementing cCMV screening as part of the newborn hearing screening in the hospital, including genetic testing, but no current protocols have been established. Yamamoto et al. (12) studied 11,900 infants born to a large group of CMV-positive mothers, finding 29.2% of the children in this population who failed their newborn hearing screening to also have cCMV. Additionally, Fowler et al. (7) completed a massive systematic review, including nearly 100,000 newborns screened for cCMV in 7 different medical centers during a 6-year period. They also propose that implementing a CMV screening protocol for all children who fail their newborn hearing screening will lead to higher rates of early identification and early intervention for these children.

If a fetus contracts CMV while in the womb, they are said to have cCMV; if they contract CMV shortly after birth, they are said to have postnatal CMV. These CMV acquisition factors should be considered when discussing the relationship between CMV and hearing loss. Of the children infected with cCMV, 90% present as asymptomatic; this percentage is likely to increase if an increased capacity for cCMV screenings in hospitals for all newborns becomes available and implemented (2). Ten percent of children with cCMV present as symptomatic, having at least one of the following conditions: hepatosplenomegaly (swelling of the liver/spleen), petechiae (small spots on skin due to bleeding), jaundice (yellowing of the skin/eyes from excessive bilirubin), and/or microcephaly (small head/skull). It is more likely for a child to be symptomatic if the mother was infected during the first trimester of pregnancy (2, 5). Symptomatic expression of cCMV increases the likelihood of neurosensory impairments, including visual impairments, by up to 60% (10) and SNHL by 44.4% (2).

#### Effects of CMV on Hearing Loss in Children

Even if a child with cCMV is born with normal hearing, a progressive SNHL can occur later in life and have adverse effects on speech development. For example, Fletcher et al. (15) found a wide variance (9–68%) in onset time of SNHL based on information from 36 previous studies. In a retrospective case study by Kim et al. (16), 11 out of 58 children (19%) with cCMV had SNHL, but the majority of them were not identified as having hearing loss (HL) during the hospital hearing screening, suggesting SNHL can occur later in life for this population even after passing a newborn hearing screening. Demmler-Harrison et al. (14) studied the long-term effects of cCMV-related HL, finding 20% of asymptomatic cCMV cases born with normal hearing to have late-onset SNHL. In a sample of 237 participants, 46% of children with primary cCMV were born with SNHL and 54% of children with primary cCMV had delayed onset SNHL, suggesting SNHL might not appear until up to 18 years of age for this population. However, in a population of 92 asymptomatic cCMV children and 51 controls, Lanzieri et al. (17) found that the risk of developing SNHL after age 5 was not significantly different than the risk for uninfected children to develop SNHL, suggesting this hearing loss is likely to manifest before entering grade school. Similarly, Salomè et al. (21) studied the long-term hearing outcomes for 102 children with asymptomatic CMV. After following up with these families for an average of 3.3 years after birth, the researchers did not find a delayed onset hearing loss for any of the children. They determined a low risk of delayed hearing impairment for children with cCMV infection who were asymptomatic during the first month of life. These contrasting research findings indicate the need for more extensive research to understand the situations in which SNHL is present with a delayed onset.

Other researchers have investigated the severity of SNHL associated with CMV. Lanzieri et al. (18) studied 168 cCMV-infected children, of which 76 were symptomatic and 92 were asymptomatic. In this population, the degree of HL was most commonly severe to profound. In a sample of 181 children with cCMV-related hearing loss, Riga et al. (19) found a significantly higher prevalence of bilateral SNHL for those with symptomatic infection compared to those with asymptomatic infection. This trend is prevalent outside of the Western world as well. Dar et al. (13) studied 1,720 Indian children who either tested positive for cCMV or failed their newborn hearing screening and found a strong correlation between the two conditions. Similarly in Belgium, Verbeeck et al. (22) evaluated 194 infants with hearing loss and found those infected with CMV before or during birth who also had hearing loss had less of a chance of recovering their hearing than uninfected children. These findings indicate that hearing loss and cCMV are strongly correlated throughout the world.

As previously noted in other texts (3, 29), prelingual SNHL can delay or limit speech and language development if not identified early. For example, Korver et al. (3) suggest there is a smaller gap in reading skills between hearing impaired children and their normal hearing peers in cases of early identification and intervention, signifying the importance of an early diagnosis. Additionally, children identified as having hearing loss before 6 months of age are predicted to have increased speech and language performance compared to children identified after 6 months of age (3). Therefore, Demmler-Harrison et al. (14) believe early identification of infants with symptomatic and asymptomatic cCMV is important for detecting SNHL and providing early monitoring and intervention.

Some researchers have also studied how medical treatment of cCMV affects the development of the auditory system. Rosenthal et al. (20) collected urine and saliva samples from 580 children with cCMV to analyze viral shedding, or the rapid replication of the virus within the child. Oftentimes, medical treatment of or vaccination against a virus will result in a shorter duration of viral shedding. These researchers found that longer duration of cCMV shedding, thus not receiving medical treatment, could predict delayed onset HL in this population. One such treatment for CMV is ganciclovir, which suddenly decreases the body's viral load to combat the infection. Some research suggests that 6 weeks of ganciclovir treatment regime drastically lowers the risk of severe hearing loss and neurological impairments within the first year of life (9, 20). In cases of symptomatic cCMV infection with central nervous system involvement, ganciclovir treatment may be indicated for 6 months, providing borderline statistically significant improvements for long-term hearing capabilities and psychomotor development (10).

#### Effects of CMV on Speech/Language Development in Children

Since speech and language development may also be limited for children with delayed physical, intellectual, and motor development, researchers have evaluated how cCMV affects these developmental milestones for this population. Lopez et al. (24) compared the intelligence, vocabulary, reading, and math abilities of cCMV-infected children with SNHL and children with normal hearing. They found significantly lower scores for the cCMV-positive group with SNHL for receptive language and full-scale intelligence measures compared to the normal hearing group. Similarly, Zhang et al. (25) examined the long-term effects of cCMV as it related to average physical and intellectual development in a rural population in Qinba, China. This area of China has a high incidence of intellectual disability and CMV in pregnancy; the researchers hypothesized CMV may contribute to intellectual and physical disabilities in this population (25). Upon further investigation, researchers discovered no significant differences between the physical development (i.e., birth weight, height, etc.) of cCMV-infected children and their uninfected peers. However, they found significantly lower IQ scores for the cCMV-infected children (25). Since intellectual capabilities are important for typical linguistic, cognitive, and social development, early identification of cCMV in children is crucial for speech and language success.

In addition, De Kegel et al. (23) examined the early motor development of children with cCMV and the deterioration of motor function for this population over time. They gathered 64 children with cCMV aged 6 months to 24 months to participate; 26 were symptomatic and 38 were asymptomatic. Of the symptomatic children, 14 had SNHL, while of the asymptomatic children, 5 had SNHL (23). This population was compared to 107 typically developing children based on gross motor performance (i.e., reflexes, stationary, locomotion, and object manipulation), fine motor performance (i.e., grasping and visual-motor integration), and balance and walking capabilities at different age groups. After analyzing the data, they found the greatest risk for gross motor delays for children with symptomatic cCMV and/or cCMV-related SNHL. Therefore, cCMV-related motor delays may impact motor speech skills development for this population.

#### Effects of CMV on Clinical Outcomes in Children

Considering the importance of speech and language development in early childhood, utilizing cochlear implants (CIs) for children with cCMV-related hearing loss is an option to mitigate the effects of decreased auditory input necessary for speech understanding. Corazzi et al. (27) compared cCMV-positive children with SNHL and children with a Connexin 26 mutation, which is a genetic mutation that is highly associated with SNHL. Both groups of children received CIs, and they found children with cCMV-related hearing loss struggled with obtaining language more than those with Connexin 26-related hearing loss. The authors stress that CIs are still a highly beneficial option for children with cCMV-related hearing loss. In France, Laccourreye et al. (29) investigated the speech of children with profound HL following cCMV infection with CIs prior to 3 years of age. They analyzed the speech perception, speech production, and speech intelligibility of 15 children both before and during 36 months of CI usage, finding both benefits and limitations to CI usage for this population. Some benefits included improved post-CI word recognition scores for closed- and open-list testing (74 and 48%, respectively), improved post-CI speech production capabilities (80% progressing beyond “meaningless vocalizations” to more complex productions), and improved speech intelligibility for 60% of the children (of which 33% could be understood by unfamiliar listeners). However, the benefits of CIs for this population may be limited depending on magnetic resonance imaging (MRI) test results. In the Laccourreye et al. (29) study, benefits were limited in instances of bilateral vestibular areflexia or brain abnormality on MRI, as demonstrated by the 53% of children never developing speech perception skills on open-list tasks, 20% only producing “meaningless vocalizations,” and 20% remaining unintelligible, despite being implanted with a CI. Therefore, most children with cCMV-related hearing loss who are implanted with CIs before 3 years of age may effectively develop speech perception, production, and intelligibility, although these benefits are limited for children with brain MRI abnormalities.

MRI can also be an important diagnostic tool when determining course of treatment for a child. Diogo et al. (28) describe the benefits of MRI as being twofold: to accurately detect fetal structural abnormalities and to provide prognostic information to the patient and family. Some MRI results can be detected during pregnancy in the developing fetus, while other MRI tests are conducted postnatally. Some key MRI abnormalities found in cCMV cases include ventricular anomalies, temporal lobe lesions, calcifications, and ventricular septations or “pseudocysts” (28). When only one of these MRI anomalies is present, cCMV is not typically suspected because other syndromes and diseases can create similar anomalies. However, when multiple lesions appear in the anterior temporal lobe, cCMV should be considered.

Cannie et al. (26) found accurate and reliable fetal MRI results predicting SNHL and neurological impairment as early as 27- and 33-week's gestation for the cCMV population, which disproved previous theories of MRI imaging being the most accurate in later stages of pregnancy. Cannie et al. (26) also classify MRI results into five categories of trauma, ranging from normal to severe: “normal; isolated frontal/parieto-occipital white matter hyperintensity; temporal/occipital cysts and/or intraventricular septa; migration disorders; and cerebellar hypoplasia and/or microcephaly” (28). These classifications of trauma are important for predicting functional outcomes for cCMV children; for example, a cCMV-infected child with normal MRI may have greater probability for normal hearing and neurologic function than a cCMV-infected child with microcephaly. Therefore, CIs may not provide adequate benefit for cCMV-infected individuals with brain lesions as described by MRI results because CIs provide maximum benefit for individuals with normal neural pathway functionality. Additionally, Natale et al. (30) discovered cases of auditory neuropathy as a result of cCMV infection. Out of 60 cCMV-infected asymptomatic children who did not undergo medical treatment, 16 children had auditory neuropathy. However, these concerns resolved as auditory thresholds spontaneously recovered over time in childhood.

Overall, cCMV is one of the most common prenatal infections causing hearing loss in a developing fetus. Whether hearing loss is present at birth or develops over time, the implications on speech and language development are evident. Since the virus also affects physical, intellectual, and motor development, care should be taken when evaluating and intervening for this population; there may be irreversible structural and functional damage to the brain that limits the benefits of CI surgery for some individuals.

### Human Immunodeficiency Virus (HIV)

HIV is a retrovirus that attacks a person's immune system. HIV-infected cells attack helper T-cells, which are crucial for developing a strong immune system, thus leaving the person immunocompromised and more susceptible to other infections (1) (Table 2). In 2006, the Centers for Disease Control and Prevention (CDC) estimated that HIV affected over “33 million people world-wide” (as cited in 31). In 2016, that number increased to 36.7 million (4). Non-Caucasian populations are disproportionately affected by HIV in the United States; in 2016, 64% of babies born with HIV in the United States were African American. (4). When a person is initially infected with HIV, the symptoms are mild (i.e., fever, headache, sore throat, etc.); however, HIV can progressively increase a person's susceptibility to contracting deadly viruses, thus becoming lethal (1). Some individuals born with HIV present no initial symptoms of an infection. Thus, the virus may go undetected for several years, especially if the child's mother is unaware of her own infection. This situation may occur, considering only 70% of people with HIV are aware of their infection (4). In the 1990's, prenatal HIV testing was developed and widely available as part of a prenatal screening that did not have to be separately requested by the patient. In 1999, Birmingham, Alabama saw an increase of testing rates from 75 to 88% due to this universal testing mechanism, thus increasing the diagnostic rates of HIV (4). Therefore, screening for HIV is crucial for timely medical intervention.

**Table 2:** Background of HIV and effects of HIV on hearing and speech and language development in children.

	**References**	**Study design**	**Population**	**Major findings**	**Search strategy**
Background (*n* = 3)	Cohen et al. (1)	Systematic Review	Individuals with virus-induced hearing loss	Many viral infections may cause congenital or acquired hearing loss.	4
	Lynch et al. (4)	Systematic Review	Several cases of maternal to child transmission of HIV	Preventing the maternal to child transmission of HIV is ideal. More research is needed for optimal early identification, intervention, and treatment. Antiretroviral therapy (ART) is recommended for this population.	4
	Woods et al. (31)	Systematic Review	Individuals with HIV-associated neurocognitive disorders (HAND)	HAND can affect motor skills, information processing, episodic memory, working memory, language, and visual perception. More research is needed to determine the best diagnostic and treatment techniques for this population.	4
Hearing loss (*n* = 8)	Dawood et al. (32)	Systematic Review	HIV-infected children from Africa, South America, North America, and Asia not treated with antiretroviral therapy (ART)	There are clear potential associations between HIV-related hearing loss and other congenital factors.	3
	Ensink et al. (33)	Systematic Review	3,491 individuals diagnosed with HIV, both children and adults	Conductive hearing loss appears to be more prevalent for children with HIV, whereas, sensorineural hearing loss appears to be more prevalent for adults with HIV compared to the general population. More studies are needed to determine if/how treatment of HIV affects these outcomes.	4
	Hrapcak et al. (34)	Cross-sectional Survey	380 HIV infected children aged 4–14 years attending an ART clinic in Lilongwe, Malawi.	Hearing loss is common in HIV infected children which shows the urgent need for improved screening tools and treatment options. Hearing loss was also found to be more common in children with frequent ear infections and ear drainage.	3
	Maro et al. (35)	Cross-sectional Survey	Cohort of HIV positive and HIV negative children from Tanzania.	Results showed HIV positive children were more likely to report dizziness and ear drainage; DPOAE levels were also lower in the HIV positive group. However, audiometric thresholds, gap detection thresholds, and auditory brainstem latencies were not found to be significantly different between the two groups.	3
	Pappas et al. (36)	Cohort Study	8 temporal bones of acquired immunodeficiency syndrome (AIDS)	HIV/AIDS may have neurotologic manifestations. Viral-like particles of HIV were detected on the tectorial membrane in 3/8 cases. Viral-like particles similar to HIV were detected in the cytoplasm of connective tissue cells. These viral-like particles may explain the neurotologic pathologies associated with HIV.	4
	Rarey (37)	Cohort Study	Individuals diagnosed with HIV with presenting otologic manifestations from 9 studies	41–71% of HIV cases are seen in the head/neck region. Otologic diagnoses related to HIV/AIDS may include varieties of hearing loss, otitis media, mastoiditis, cholesteatoma, and/or tympanic membrane perforation. Many tests must be completed to pinpoint the exact relationship between HIV and these otologic manifestations.	4
	Torre et al. (38)	Correlational/Cohort Study	145 HIV+ children/adolescents age 7–16 years and 86 HIV-exposed but unaffected children/adolescents age 7–16 years	Hearing loss is more common for HIV+ and HIV-exposed but unaffected children than children not exposed to HIV. Hearing loss risks may increase as severity of HIV+ infection increases.	3
	Torre et al. (39)	Cohort Study	Children born from HIV positive mothers and aged 7–16 years.	HIV infection was not found to be associated with poorer distortion product otoacoustic emissions; cochlear function was similar between the two groups. Positive HIV children with higher viral loads had worse cochlear function.	3
Speech/ Language (*n* = 13)	Alcock et al. (40)	Comparative Study	Children exposed to HIV in *utero* in rural Kenya	Older HIV exposed uninfected children had poorer language skills compared to the controls. HIV positive children scored more poorly compared to the controls. The results show HIV infection is related to early language development.	3
	Benki-Nugent et al. (41)	Cohort Study	HIV infected and HIV uninfected infants	HIV infected infants with viral suppression on antiretroviral therapy were found to have better developmental milestones; deficits were worse compared to uninfected infants.	3
	Boivin et al. (42)	Cohort Study	14 asymptomatic HIV-infected Zairian children compared to 20 HIV-negative children and 11 children in the control group	Quantitative, verbal, and memory deficits were observed in the asymptomatic HIV-positive children. The risk of these deficits increases with the presence of brain abnormalities/neurological impairment. HIV impairs motor and spatial memory development in the central nervous system.	3
	Brackis-Cott et al. (43)	Cohort Study	340 youths between 6 and 16 years of age (206 HIV positive and 134 HIV negative children) and their caregivers. The children were either perinatally exposed and infected or exposed and uninfected.	HIV status was associated with PPVT-III and WRAT-3 standard scores. The results showed that poor language ability was common in HIV+ unaffected youths as well as those affected by HIV.	3
	Brahmbhatt et al. (44)	Cohort Study	329 mothers and children aged 0–6 years	HIV positive children were more likely to have deficits in neurodevelopment including in receptive and expressive language. Findings show early diagnosis and treatment of HIV in children should be a priority.	3
	DeCarlie et al. (45)	Qualitative Analysis	100 CT images of children with untreated AIDS	Cerebral calcification was found in the samples of prenatally-infected HIV patients with encephalopathy. Abnormalities of the cerebellum are seen at a high percentage in children with HIV.	4
	Redmond et al. (46)	Cohort Study	Perinatally-acquired HIV youth and perinatally exposed but uninfected youth	The results showed the youth exposed to HIV (both infected and uninfected) are at risk for language impairment. Family history of language delays was also shown to be a risk factor for persistent issues.	3
	Rice et al. (47)	Comparative Study	Children aged 7–16 perinatally infected with HIV and HIV exposed but uninfected	Children who are perinatally exposed are at higher risk for language impairment, however this risk was not increased for children with HIV.	3
	Rice et al. (48)	Cohort Study	Preschool aged monolingual perinatally HIV exposed, yet uninfected, children in the United States.	Risk of language impairment was higher in HIV exposed children when compared to the norm values. The risk for speech impairments was not elevated.	3
	Ntozini et al. (49)	Observational sub-study of a cluster-randomized trial	Children who are HIV exposed uninfected and children who were not exposed to HIV (pregnant women)	At 2 years of age, child development and vocabulary scores were about 0.15 standard deviations less in the group of children exposed to HIV compared to the HIV-unexposed group.	3
	Van Rie et al. (50)	Comparative Study	35 HIV infected children, 35 HIV affected children, and 90 control children; aged 18 to 72 months.	Young HIV infected children were found to need the earliest intervention. The study showed 60% of HIV infected children had severe delay in cognitive function, 85% had delays in language expression, and 77% had delays in language comprehension.	3
	Wolters et al. (51)	Cohort Study	36 children age 1–10 years old with symptomatic HIV infection compared to 20 uninfected siblings	Expressive language more impaired than receptive language. Greater abnormality based on CT scan was correlated with poorer receptive and expressive language abilities. The uninfected siblings did not have significantly lower language scores.	3
	Wolters et al. (52)	Longitudinal Study	Same population as Wolters et al. (51)	24 months after the original study, language scores declined significantly despite antiretroviral therapy. Cognitive functioning remained stable. CT scans did not change during the 24-month time period.	3

*CT, computerized tomography; DPOAE, distortion product otoacoustic emissions; HIV, human immunodeficiency virus*.

#### Effects of HIV on Hearing Loss in Children

In 14 to 49% of cases, HIV can affect the auditory system, resulting in hearing loss, tinnitus, facial nerve palsy, chronic otitis media, and malignancies or cancers (1). HIV-related hearing loss can be caused directly (i.e., as a direct result of HIV infection) or indirectly (i.e., due to increased susceptibility to other infections or ototoxic medications used to treat other diseases resulting from HIV). While hearing loss in HIV-infected individuals is more common later in life (i.e., post-lingual hearing loss), infants can present with hearing loss after direct infection or exposure *in-utero* (1, 38). The types of hearing loss present in this population widely vary, including progressive or sudden onset, unilateral or bilateral manifestations, and conductive, sensorineural, or mixed. Since HIV greatly affects a child's developing immune system, children with HIV are more likely to have a conductive hearing loss rather than sensorineural due to increased susceptibility to infections and complications such as otitis externa, otitis media, cholesteatoma, acquired aural atresia, aural polyps, and malignancy (1, 32, 33). For example, Hrapcak et al. (34) completed a cross-sectional study in Malawi on 380 HIV-infected children aged 4–14 years old. Of the 23% of children who had hearing loss, 82% had a conductive hearing loss component. Similarly, Maro et al. (35) compared 131 HIV+ children to 113 HIV- children in Tanzania. They found the HIV+ children to be more likely to have ear drainage and abnormal tympanometry results, likely indicating a higher prevalence of middle ear disorders or infections.

HIV may also affect other portions of the peripheral auditory system. Research on distortion product otoacoustic emissions (DPOAEs), which are a measure of the function of the outer hair cells within the cochlea, has been mixed. In addition to finding higher prevalence of middle ear disorders, Maro et al. (35) found HIV+ children to have abnormal DPOAEs. However, Torre et al. (39) compared 89 HIV-infected children to 83 HIV-exposed but uninfected children, finding no correlation between HIV infection and DPOAEs. These contrasting research findings indicate the need for more research to define the circumstances in which the peripheral auditory system acquires damage related to HIV. Additionally, in a systematic review of HIV and hearing loss in children, unilateral hearing loss seemed to be more correlated with HIV than bilateral hearing loss (32). Infants infected with HIV can also develop SNHL later in life, contributing to the prevalence of mixed hearing losses for children with HIV-related HL. HIV has been detected in portions of the inner ear, including hair cells of both the auditory and vestibular system, stria vascularis cells that supply nutrients to the cochlea, and in the tectorial membrane, which moves in response to fluid movement within the cochlea (1, 36).

In addition to its effects on the peripheral auditory system, HIV can also affect the functionality of the central auditory nervous system. Rarey (37) discovered people who have HIV often present with abnormal auditory brainstem response (ABR) results, suggesting anomalies within the auditory nerve and other portions of the central auditory system. Although HIV has been shown to affect the auditory system, either directly or indirectly, the mechanisms by which this is accomplished are widely unknown. Since HIV causes individuals to be more susceptible to other diseases that can impact the peripheral and central auditory system, determining whether the hearing loss was caused by HIV or by the other diseases is ambiguous. Therefore, MRI results, in combination with medical history, may be required to pinpoint the exact cause of hearing loss in HIV patients.

#### Effects of HIV on Speech/Language Development in Children

Previous research has highlighted a common trend of deteriorated language development for prenatal HIV-infected children and prenatal HIV-exposed but uninfected children. Alcock et al. (40) examined the prevalence of language disorders for 18 HIV-infected children and 14 HIV-exposed but uninfected children in Kenya. They found the infected children had language delays compared to a population of unexposed and uninfected controls, while the exposed but uninfected children demonstrated relatively typical language development compared to the controls until older childhood, when language development generally becomes more complex. These results suggest markedly different language development for children with HIV-infection or exposure when compared to uninfected and unexposed children. Similarly, Ntozini et al. (49) compared the early child development of HIV-exposed children and HIV-unexposed children in Zimbabwe, finding the exposed children scored 0.15 standard deviations below the unexposed group by 2 years of age.

Both HIV-infected infants and HIV-exposed but uninfected infants may also have speech or language delays in preschool years. Rice et al. (48) studied preschool children throughout the United States and Puerto Rico who were either HIV-infected at birth or HIV-exposed but uninfected at birth. They also studied the effect of *in-utero* antiretroviral therapy (ART, a medical treatment for HIV) for this population and its effect on speech and language development. They estimated the prevalence of language impairment to be 15%, but this prevalence was greatly reduced when the infant was exposed to ART *in-utero*. However, the risk for speech impairment was not elevated for this population. Similarly, Rice et al. (47) found out of 468 school-age children with perinatal HIV infection, 39% had a language impairment. These results suggest a higher prevalence of language impairments than speech impairments for this population.

These language impairments may also extend into the early adolescent years. Redmond et al. (46) completed additional testing on the children from the Rice et al. (47) study to determine if these language impairments continued into early adolescence. They found approximately one third of the infants born to HIV-positive mothers had language impairments that persisted into adolescence (46). Additionally, Brackis-Cott et al. (43) studied the receptive language and word recognition abilities of 206 perinatally HIV-infected youth aged 9–16 years old. They found 60% of this population scored below the 25th percentile for receptive language testing, and 39% scored below the 10th percentile. For word recognition testing, 49% scored below the 25th percentile and 28% scored below the 10th percentile. Although socioeconomic status and demographic characteristics may also contribute to these disparities, this research suggests that HIV-infection as an infant may impact the language development of these children as they progress into adolescence.

Anatomically, HIV can affect parts of the central nervous system (CNS) responsible for speech and language development. Through neuroimaging techniques, DeCarlie et al. (45) found characteristic brain abnormalities in HIV-infected children, including “cortical atrophy, white matter hypodensity, and basal ganglia calcifications” (52). These findings relate to clinical presentations of language deficits. Wolters et al. (51) compared the expressive and receptive language skills of HIV-infected children to their uninfected siblings, finding expressive language deficits more prevalent in HIV-infected children (52). This provided a definite link between HIV infection and CNS abnormalities, absent from other environmental factors. However, it was unclear when these deficits first appeared. Further research is required to understand the onset of these expressive language deficits and to adequately identify whether these children would benefit from speech language therapy.

HIV can also interfere with typical cognitive, motor and neurodevelopment, all of which are crucial for speech and language development. In a sample of 35 HIV-infected preschoolers and 35 HIV-exposed preschoolers in the Democratic Republic of Congo, Van Rie et al. (50) found a high prevalence of language expression deficits (85%), language comprehension deficits (77%), cognitive functioning delays (60%), and motor development delays (29%) for this population of children. Additionally, Boivin et al. (42) compared the performance of HIV-positive children to HIV-negative children in Zairia, Nigeria. HIV-positive children were defined as children born to HIV-positive mothers and testing positive themselves upon birth. HIV-negative children were defined as children born to HIV-positive mothers but testing negative themselves upon birth. These groups were compared to a control group, which included children born to HIV-negative mothers and testing negative themselves upon birth. The HIV-positive children scored significantly lower on social, language, and gross motor development compared to the HIV-negative and control groups, suggesting symptomatic expression of HIV is a potential contributing factor to these delays (42). They also found HIV-infected children to have spatial memory deficits compared to uninfected children. Unfortunately, many children in the HIV-positive group passed away before a follow-up study was conducted due to the devastating lethal effects of HIV, so a larger sample size should be considered for maximum generalizability.

Some research has considered how medical treatment of HIV impacts the duration of neurological developmental delays. Brahmbhatt et al. (44) studied 329 mother-infant pairs in Uganda. They found HIV-positive children who underwent ART (antiretroviral therapy) for 24–60 months had decreased impairments in receptive and expressive language, as well as fine motor skills, compared to HIV-positive children who underwent ART for < 12 months. Those with longer ART duration showed deficits compared to HIV-negative unexposed children, but these deficits were not as severe compared to those with less ART duration. Similarly, Benki-Nugent et al. (41) compared the developmental milestones of children with ART therapy to HIV-unexposed children. They found children with ART still showed delayed milestones for sitting with and without support, walking with and without support, monosyllabic speech, and throwing toys. Therefore, it is recommended that children begin ART early to mitigate some of these developmental delays.

#### Effects of HIV on Clinical Outcomes in Children

While there is limited research on how CIs or hearing aids assist children with HIV-related hearing loss, fitting this population with assistive listening technologies may be challenging. Since children with HIV typically present with conductive hearing losses (usually caused by complications of other infections) more often than sensorineural, it may be difficult for these children to wear hearing aids due to inflammation, pain, and drainage in the external auditory meatus. For those with sensorineural hearing loss, CI surgery can be very risky because HIV greatly weakens a person's immune system; CI surgery can introduce other infections into the inner ear space, possibly leading to more serious complications. Therefore, Cohen et al. (1) recommend only conducting CI surgery in patients with a stable amount of immune system cells, decreasing the likelihood of introducing diseases that would be difficult to combat.

Overall, HIV can have life-threatening implications due to increased risk of contracting other diseases. If not recognized at birth, hearing, motor, cognitive, and language development can be delayed or limited as the disease progresses. While hearing loss and speech developmental delays are not the most pressing concerns for this population, they are important elements of a child's quality of life that should also be considered.

### CMV and HIV

There has been limited research on the effects of CMV and HIV together on speech, language, and hearing development (Table 3). A potential relationship may exist for children with prenatal HIV who are subsequently exposed to CMV due to a weakened immune system. Adachi et al. (53) collected urine samples of 992 infants born to mothers with HIV who were not receiving treatment. They found that “cCMV was present in 23.2% of infants with *in utero*” HIV infection, suggesting a relatively high rate of CMV infection in HIV-exposed infants. Purswani et al. (54) studied a similar population, except the mothers with HIV were receiving ART. Of 895 mothers with HIV-exposed children, 90% were receiving ART. Eight of these infants were diagnosed with cCMV, suggesting a prevalence of 0.89% for cCMV and HIV when receiving treatment. Although the prevalence of cCMV is much lower for HIV-exposed children when their mothers receive therapy, this prevalence rate is still higher than that of the general population, so HIV-exposed children still have a higher risk of contracting cCMV than an unexposed child.

**Table 3:** Background of both CMV and HIV in children.

	**References**	**Study design**	**Population**	**Major findings**	**Search strategy**
Background (*n* = 2)	Adachi et al. (53)	Clinical Randomized Control Trial	1,684 HIV-infected pregnant women from Brazil, South Africa, Argentina, and the United States; infants tested positive for HIV at birth and at regular intervals up to 6 months old	Of the 992/1,684 newborns with urine samples, 6.5% had detectable CMV in their urine. The rate of cCMV among HIV-infected infants was 18%. Of these, 23.2% had “*in-utero*” initial infection, and 9.1% had “intrapartum” initial infection. These rates were highest for mothers not receiving antiretroviral drug therapy during pregnancy.	1
	Purswani et al. (54)	Cohort Study	895 HIV-exposed but uninfected children at 22 sites in Puerto Rico and the US	8 infants who were HIV-exposed but uninfected tested positive for cCMV, with a projected prevalence of 1.2–1.3% after sensitivity adjustments. There were no noted differences in cognition, language, and hearing assessments for these children.	1

*cCMV, congenital cytomegalovirus; CMV, cytomegalovirus; HIV, human immunodeficiency virus*.

It is unclear how the combination of cCMV and HIV affect hearing, speech, and language development for these children. One may hypothesize since each disease independently may affect these developments, the combination of these diseases may result in more severe delays and abnormalities. More research is needed to specifically quantify these effects on hearing, speech, and language development, but individuals should consider early identification and treatment of these diseases to mitigate the potentially negative effects of having both infections simultaneously.

## Conclusion

Both cCMV and congenital HIV can adversely affect a child's hearing, speech, and language development. These infections can create structural and functional anomalies in the peripheral and central auditory systems, as well as the central nervous system, which may limit the benefits of CI and hearing aid fittings. MRI and other neuroimaging techniques can be helpful in diagnosing and treating hearing loss in a child with one of these diseases by determining whether they would be a good candidate for hearing devices based on anatomical findings. Although CI surgery generally presents risk for patients, this risk may be amplified for children with cCMV and HIV due to their weakened immune systems. Speech and language therapy may also be warranted for children with notable speech and language performance deviations.

One of the best methods for limiting the devastating effects of cCMV and HIV is early identification. If pregnant mothers are screened early in pregnancy for CMV and HIV, adequate medical treatment options can be readily available for their developing babies and themselves. Since these infections can be life-threatening, hearing and speech development may be one of the last concerns these families and healthcare providers have for the child. However, if the child is in a stable condition and progressing well medically, aiding the hearing loss and offering speech and language therapy may greatly improve the child's quality of life.

## Author Contributions

All authors reviewed the papers, analyzed the data, wrote the article, and discussed the results at all states.

## Funding

This work was supported by an internal funding source from the UF College of Public Health and Health Professions.

## Conflict of Interest

The authors declare that the research was conducted in the absence of any commercial or financial relationships that could be construed as a potential conflict of interest.

## Publisher's Note

All claims expressed in this article are solely those of the authors and do not necessarily represent those of their affiliated organizations, or those of the publisher, the editors and the reviewers. Any product that may be evaluated in this article, or claim that may be made by its manufacturer, is not guaranteed or endorsed by the publisher.
